# Poly[μ_3_-chlorido-μ_2_-chloridodichlorido(μ-dimethyl sulfoxide-κ^2^ *O*:*S*)(dimethyl sulfoxide-κ*O*)(μ-pyrimidine-κ^2^ *N*:*N*′)­ruthenium(III)sodium]

**DOI:** 10.1107/S1600536811017211

**Published:** 2011-05-14

**Authors:** Skylar Ferrara, Ava Kreider-Mueller, Joseph M. Tanski, Craig M. Anderson

**Affiliations:** a Department of Chemistry, Bard College, Annandale-on-Hudson, NY 12504, USA; b Department of Chemistry, Vassar College, Poughkeepsie, NY 12604, USA

## Abstract

The title complex, [NaRuCl_4_(C_4_H_4_N_2_)(C_2_H_6_OS)_2_]_*n*_, is the sodium salt of monoanionic octa­hedral [Ru^III^Cl_4_(pyrimidine)(DMSO)]^−^ in which the sulfur-bound dimethyl sulfoxide (DMSO) and pyrimidine ligand are oriented *trans* to one another on the Ru^III^ atom. The average of the four Ru—Cl bond lengths is 2.355 (15) Å, and the Ru—S and Ru—N bond lengths are 2.2853 (3) and 2.1165 (11) Å, respectively. The complex forms a chain, with a six-coordinate sodium ion bridging the ruthenium(III) units. The sodium cation is coordinated by *cis*-chloride ligands on ruthenium [Na—Cl = 2.9576 (7) and 2.6988 (7) Å], chloride and DMSO ligands from the ruthenium complexes related by inversion [Na—Cl and Na—O = 2.8888 (7) and 2.2623 (12) Å, respectively], a nitro­gen ligand from the pyrimidine of the tetrachlorido­ruthenium(III) complex related by the twofold rotation axis [Na—N = 2.5224 (14) Å] and an oxygen-bound DMSO [Na—O = 2.3165 (12) Å].

## Related literature

For general background to ruthenium complexes as anti-cancer agents, see: Kostova (2006 ▶); Antonarakis & Emadi (2010 ▶); Silva (2010 ▶). For the synthesis of related complexes and precursors, see: Alessio *et al.* (1991 ▶, 1993 ▶); Jaswal *et al.* (1990 ▶). For related structures with the tetra­chloro ruthenium (III) motif and electron-withdrawing ligand, see: Alessio *et al.* (1995 ▶); Anderson & Beauchamp (1995 ▶). For related multi-nuclear species, see: Herman *et al.* (2008 ▶); Iengo *et al.* (1999 ▶). For a very closely related structure with pyrazine in place of pyrimidine, showing a very similar network bonding, see: Anderson *et al.* (2007 ▶).
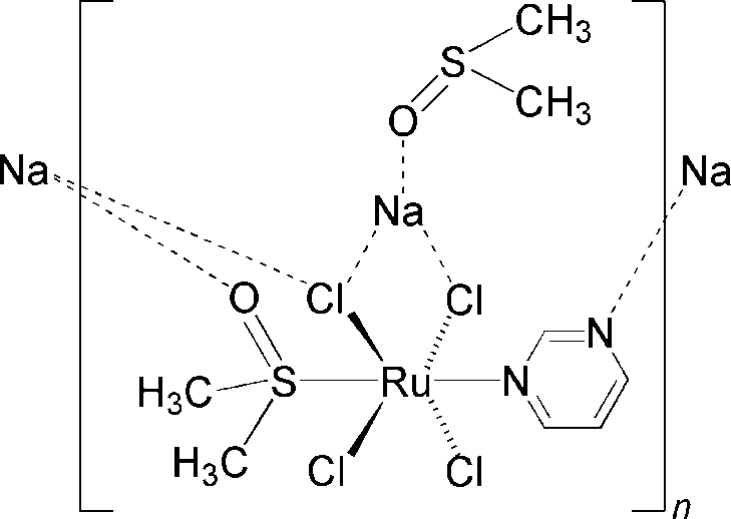


## Experimental

### 

#### Crystal data

[NaRuCl_4_(C_4_H_4_N_2_)(C_2_H_6_OS)_2_]
*M* *_r_* = 502.21
Monoclinic, 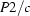

*a* = 12.5052 (6) Å
*b* = 10.9917 (5) Å
*c* = 13.1837 (6) Å
β = 91.680 (1)°
*V* = 1811.37 (15) Å^3^
*Z* = 4
Mo *K*α radiation
μ = 1.71 mm^−1^
*T* = 125 K
0.25 × 0.23 × 0.10 mm

#### Data collection

Bruker APEXII CCD diffractometer
Absorption correction: multi-scan (*SADABS*; Bruker, 2007 ▶) *T*_min_ = 0.675, *T*_max_ = 0.848
25088 measured reflections
5227 independent reflections
4922 reflections with *I* > 2σ(*I*)
*R*_int_ = 0.018

#### Refinement

*R*[*F* ^2^ > 2σ(*F* ^2^)] = 0.018
*wR*(*F* ^2^) = 0.045
*S* = 1.07
5227 reflections
185 parameters
H-atom parameters constrained
Δρ_max_ = 0.61 e Å^−3^
Δρ_min_ = −0.87 e Å^−3^

### 

Data collection: *APEX2* (Bruker, 2007 ▶); cell refinement: *SAINT* (Bruker, 2007 ▶); data reduction: *SAINT*; program(s) used to solve structure: *SHELXS97* (Sheldrick, 2008 ▶); program(s) used to refine structure: *SHELXL97* (Sheldrick, 2008 ▶); molecular graphics: *SHELXTL* (Sheldrick, 2008 ▶); software used to prepare material for publication: *SHELXTL*.

## Supplementary Material

Crystal structure: contains datablocks I, global. DOI: 10.1107/S1600536811017211/om2427sup1.cif

Structure factors: contains datablocks I. DOI: 10.1107/S1600536811017211/om2427Isup2.hkl

Additional supplementary materials:  crystallographic information; 3D view; checkCIF report

## Figures and Tables

**Fig. 1.**
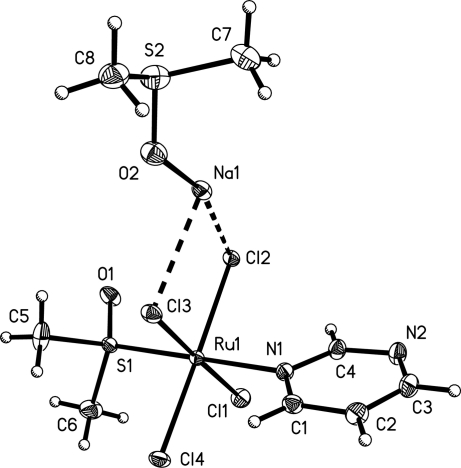
A view of the title compound, with displacement ellipsoids shown at the 50% probability level.

**Fig. 2.**
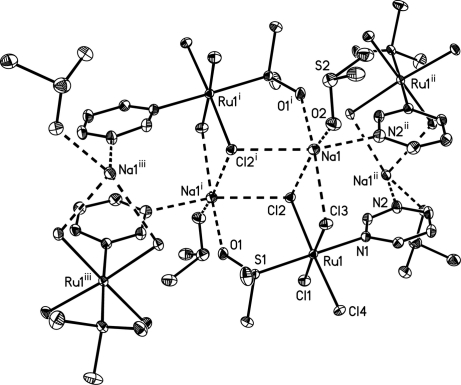
A view of the one-dimensional chain with displacement ellipsoids shown at the 50% probability level. Hydrogen atoms have been removed for clarity. [Symmetry codes: (i) -x + 1, -y, -z + 1; (ii) -x + 1, y, -z + 1/2; (iii) x, -y, z + 1/2].

## supplementary crystallographic information

### Comment

Ruthenium complexes are thought to be good candidates for the next generation of metal-based anti-cancer agents following the very successful platinum complexes whose most famous example is cisplatin; platinum complexes struggle with general toxicity, drug resistance, and lack of water solubility (Kostova, 2006; Antonarakis & Emadi, 2010; Silva, 2010). For synthesis of ruthenium precursors see: Alessio *et al.* (1991) and Jaswal *et al.* (1990).

The title complex exhibits a nearly octahedral coordination geometry at ruthenium, and the dimethyl sulfoxide (DMSO) coordinated sodium cation is associated with the complex *via cis-*chloride ligands (Fig. 1). The sulfur-bound DMSO and pyrimidine ligands are *trans*, with Ru1—S1 and Ru1—N1 bond distances of 2.2853 (3)Å and 2.117 (1) Å, respectively, and an average Ru1—Cl distance of 2.35 (2) Å. The Ru1—Cl, Ru1—S1 and Ru1—N1 distances are very similar to those found in three related RuCl_4_(DMSO)^-1^ structures containing the aromatic nitrogen donor ligand pyrazine, [Na][RuCl_4_(pyrazine)(DMSO)] (Anderson *et al.*, 2007), Na_2_[*trans,cis,trans*-Ru^III^Cl_4_(DMSO)(µ-pyrazine)]_2_Pt^II^Cl_2_, and (tetraphenylphospohium)_2_[*trans,trans,trans*-Ru^III^Cl_4_(DMSO)(µ-pyrazine)]_2_Pt^II^Cl_2_ (Herman *et al.*, 2008). The Ru—Cl bond distances are also similar to other Ru(III) tetrachloro complexes containing electron withdrawing ligands such as CO (Alessio *et al.*, 1995) or nitroimidazole (Anderson & Beauchamp, 1995). The dimeric species Na_2_[*trans,trans*-Ru^III^Cl_4_(DMSO)]_2_(µ-pyrmidine)] (Iengo *et al.*, 1999) and the monomeric species Na[*trans-*Ru^III^Cl_4_(DMSO)(imidazole)] (Alessio *et al.*, 1993) show a similar coordination around the ruthenium centers.

The six-coordinate, distorted octahedral sodium cation acts to bridge the ruthenium complex into an infinite one-dimensional chain (Fig. 2). On each Ru center, *cis-*chloride ligands coordinate to sodium with Na1—Cl2 and Na1—Cl3 distances of 2.9576 (7)Å and 2.6988 (7) Å, respectively. A centrosymmetric dimer is formed by additional sodium coordination to Cl2^i^ and the DMSO oxygen O1^i^ of the neighboring ruthenium complex, with Na1—Cl2^i^ distance of 2.8888 (7)Å and Na1—O1^i^ distance of 2.262 (1) Å. The dimer is linked into an infinite one-dimensional chain by further coordination of the sodium ion to nitrogen N2^ii^ of the pyrimidine lignd of the ruthenium complex related by the twofold rotation axis with a Na1—N2^ii^ bond distance of 2.522 (1) Å. The final coordination site of the octahedral sodium ion is occupied by a molecule of DMSO, with Na1—O2 length 2.317 (1) Å. This infinite one-dimensional chain with the sodium cation acting as a bridge is similar to the related structure [Na][RuCl_4_(pyrazine)(DMSO)] (Anderson *et al.*, 2007), the difference being the slight twist observed in the pyrimidine complex due to the *meta* nitrogen donor atoms as opposed to a more linear case for the pyrazine complex with the *para* nitrogen donor atoms.

### Experimental

The title complex was prepared by mixing a solution of [Na][RuCl_4_(DMSO)_2_] in acetone/DMSO (10:1) with an acetone solution of pyrimidine in fivefold excess. The resulting solution was mixed for several hours and then placed at 5 °C for several days. Large block-like crystals appeared; one was taken for X-ray diffraction studies. The others were used for spectroscopic characterization. For similar spectroscopic results see Anderson *et al.*, (2007). Selected IR (cm^-1^): 1596 (pyrimidine), 1085 s (DMSO-*S*), 431 m (Ru-*S*). Selected ^1^H NMR: (D_2_O/p.p.m.): -13.9 (br, DMSO-*S*), 5.15 (pyrimidine H); -0.85 (br, pyrimidine H).

### Refinement

All non-hydrogen atoms were refined anisotropically. Hydrogen atoms on carbon were included in calculated positions and refined using a riding model at C–H = 0.95 or 0.98 Å and *U*_iso_(H) = 1.2 or 1.5 × *U*_eq_(C) of the aryl and methyl C-atoms, respectively. The extinction parameter (EXTI) refined to zero and was removed from the refinement.

### Crystal data

**Table:**

[NaRuCl_4_(C_4_H_4_N_2_)(C_2_H_6_OS)_2_]	*F*(000) = 996
*M**_r_* = 502.21	*D*_x_ = 1.842 Mg m^−^^3^
Monoclinic, *P*2/*c*	Mo *K*α radiation, λ = 0.71073 Å
Hall symbol: -P 2yc	Cell parameters from 9970 reflections
*a* = 12.5052 (6) Å	θ = 2.5–30.4°
*b* = 10.9917 (5) Å	µ = 1.71 mm^−^^1^
*c* = 13.1837 (6) Å	*T* = 125 K
β = 91.680 (1)°	Block, orange
*V* = 1811.37 (15) Å^3^	0.25 × 0.23 × 0.10 mm
*Z* = 4	

### Data collection

**Table:**

Bruker APEXII CCD diffractometer	5227 independent reflections
Radiation source: fine-focus sealed tube	4922 reflections with *I* > 2σ(*I*)
graphite	*R*_int_ = 0.018
φ and ω scans	θ_max_ = 30.5°, θ_min_ = 1.6°
Absorption correction: multi-scan (*SADABS*; Bruker, 2007)	*h* = −17→17
*T*_min_ = 0.675, *T*_max_ = 0.848	*k* = −15→15
25088 measured reflections	*l* = −17→17

### Refinement

**Table:**

Refinement on *F*^2^	Primary atom site location: structure-invariant direct methods
Least-squares matrix: full	Secondary atom site location: difference Fourier map
*R*[*F*^2^ > 2σ(*F*^2^)] = 0.018	Hydrogen site location: inferred from neighbouring sites
*wR*(*F*^2^) = 0.045	H-atom parameters constrained
*S* = 1.07	*w* = 1/[σ^2^(*F*_o_^2^) + (0.0191*P*)^2^ + 1.3448*P*] where *P* = (*F*_o_^2^ + 2*F*_c_^2^)/3
5227 reflections	(Δ/σ)_max_ = 0.003
185 parameters	Δρ_max_ = 0.61 e Å^−^^3^
0 restraints	Δρ_min_ = −0.87 e Å^−^^3^

### Special details

**Table:**

Experimental. A suitable crystal was mounted in a nylon loop with Paratone-N cryoprotectant oil and data was collected on a Bruker *APEX* II CCD platform diffractometer. The structure was solved using direct methods and standard difference map techniques, and was refined by full-matrix least-squares procedures on F^2^ with *SHELXTL* Version 6.14 (Sheldrick, 2008).
Geometry. All e.s.d.'s (except the e.s.d. in the dihedral angle between two l.s. planes) are estimated using the full covariance matrix. The cell e.s.d.'s are taken into account individually in the estimation of e.s.d.'s in distances, angles and torsion angles; correlations between e.s.d.'s in cell parameters are only used when they are defined by crystal symmetry. An approximate (isotropic) treatment of cell e.s.d.'s is used for estimating e.s.d.'s involving l.s. planes.
Refinement. Refinement of *F*^2^ against ALL reflections. The weighted *R*-factor *wR* and goodness of fit *S* are based on *F*^2^, conventional *R*-factors *R* are based on *F*, with *F* set to zero for negative *F*^2^. The threshold expression of *F*^2^ > σ(*F*^2^) is used only for calculating *R*-factors(gt) *etc*. and is not relevant to the choice of reflections for refinement. *R*-factors based on *F*^2^ are statistically about twice as large as those based on *F*, and *R*- factors based on ALL data will be even larger.

### Fractional atomic coordinates and isotropic or equivalent isotropic displacement parameters (Å^2^)

**Table:**

	*x*	*y*	*z*	*U*_iso_*/*U*_eq_	
Ru1	0.710023 (8)	0.198037 (9)	0.478554 (8)	0.01123 (3)	
Cl1	0.85124 (3)	0.11358 (3)	0.38991 (3)	0.01888 (7)	
Cl2	0.58872 (3)	0.05284 (3)	0.41162 (2)	0.01429 (6)	
Cl3	0.56492 (3)	0.28961 (3)	0.56229 (3)	0.01702 (7)	
Cl4	0.81971 (3)	0.35246 (3)	0.54845 (3)	0.01820 (7)	
S1	0.75569 (3)	0.07509 (3)	0.61274 (2)	0.01419 (6)	
S2	0.15245 (3)	0.26924 (4)	0.56476 (3)	0.02733 (9)	
Na1	0.39260 (5)	0.17477 (5)	0.47975 (4)	0.01674 (11)	
O1	0.72781 (9)	−0.05555 (9)	0.60442 (8)	0.0226 (2)	
O2	0.27052 (9)	0.29182 (10)	0.56554 (9)	0.0234 (2)	
N1	0.67216 (9)	0.30751 (10)	0.35040 (9)	0.0143 (2)	
N2	0.63506 (11)	0.31171 (11)	0.17229 (9)	0.0190 (2)	
C1	0.65932 (11)	0.42887 (13)	0.35492 (11)	0.0172 (3)	
H1B	0.6663	0.4691	0.4185	0.021*	
C2	0.63608 (12)	0.49591 (13)	0.26817 (12)	0.0209 (3)	
H2B	0.6289	0.5819	0.2706	0.025*	
C3	0.62363 (12)	0.43273 (13)	0.17765 (11)	0.0207 (3)	
H3A	0.6064	0.4767	0.1173	0.025*	
C4	0.66019 (11)	0.25468 (13)	0.25867 (10)	0.0167 (3)	
H4A	0.6706	0.1692	0.2554	0.020*	
C5	0.70393 (15)	0.12935 (16)	0.72786 (12)	0.0282 (3)	
H5A	0.7338	0.0815	0.7846	0.042*	
H5B	0.6258	0.1216	0.7256	0.042*	
H5C	0.7235	0.2150	0.7371	0.042*	
C6	0.89514 (13)	0.07824 (16)	0.64379 (13)	0.0270 (3)	
H6A	0.9099	0.0267	0.7032	0.041*	
H6B	0.9172	0.1620	0.6591	0.041*	
H6C	0.9351	0.0478	0.5862	0.041*	
C7	0.10238 (14)	0.30545 (16)	0.44103 (14)	0.0286 (3)	
H7A	0.1303	0.2470	0.3923	0.043*	
H7B	0.1252	0.3877	0.4229	0.043*	
H7C	0.0241	0.3016	0.4396	0.043*	
C8	0.09779 (13)	0.39468 (17)	0.63121 (13)	0.0280 (3)	
H8A	0.1257	0.3946	0.7015	0.042*	
H8B	0.0196	0.3877	0.6307	0.042*	
H8C	0.1179	0.4707	0.5980	0.042*	

### Atomic displacement parameters (Å^2^)

**Table:**

	*U*^11^	*U*^22^	*U*^33^	*U*^12^	*U*^13^	*U*^23^
Ru1	0.01237 (5)	0.01042 (5)	0.01084 (5)	−0.00154 (3)	−0.00045 (3)	−0.00023 (3)
Cl1	0.01630 (15)	0.02092 (16)	0.01953 (15)	0.00148 (12)	0.00268 (11)	−0.00202 (12)
Cl2	0.01616 (14)	0.01303 (13)	0.01361 (13)	−0.00274 (11)	−0.00093 (10)	−0.00138 (11)
Cl3	0.01559 (14)	0.01744 (15)	0.01805 (15)	−0.00118 (11)	0.00076 (11)	−0.00633 (11)
Cl4	0.01779 (15)	0.01504 (14)	0.02153 (16)	−0.00515 (11)	−0.00358 (12)	−0.00077 (12)
S1	0.01623 (15)	0.01305 (14)	0.01310 (14)	−0.00307 (11)	−0.00283 (11)	0.00139 (11)
S2	0.02289 (19)	0.02494 (19)	0.0341 (2)	−0.00204 (15)	−0.00019 (15)	0.00201 (16)
Na1	0.0173 (3)	0.0158 (3)	0.0170 (3)	−0.0015 (2)	−0.0024 (2)	−0.0016 (2)
O1	0.0303 (6)	0.0128 (5)	0.0239 (5)	−0.0053 (4)	−0.0103 (4)	0.0034 (4)
O2	0.0188 (5)	0.0248 (6)	0.0265 (6)	0.0019 (4)	−0.0004 (4)	−0.0036 (4)
N1	0.0159 (5)	0.0127 (5)	0.0141 (5)	−0.0003 (4)	−0.0005 (4)	0.0009 (4)
N2	0.0240 (6)	0.0179 (6)	0.0152 (6)	0.0009 (5)	−0.0002 (5)	0.0027 (4)
C1	0.0183 (6)	0.0142 (6)	0.0188 (6)	−0.0015 (5)	−0.0015 (5)	−0.0007 (5)
C2	0.0245 (7)	0.0137 (6)	0.0242 (7)	−0.0001 (5)	−0.0026 (6)	0.0036 (5)
C3	0.0246 (7)	0.0178 (7)	0.0195 (7)	0.0012 (5)	−0.0019 (5)	0.0062 (5)
C4	0.0217 (7)	0.0139 (6)	0.0144 (6)	0.0000 (5)	0.0007 (5)	0.0007 (5)
C5	0.0424 (10)	0.0282 (8)	0.0140 (7)	0.0057 (7)	0.0034 (6)	0.0030 (6)
C6	0.0187 (7)	0.0309 (8)	0.0309 (8)	−0.0042 (6)	−0.0096 (6)	0.0104 (7)
C7	0.0231 (8)	0.0313 (9)	0.0312 (9)	−0.0011 (6)	−0.0048 (6)	−0.0054 (7)
C8	0.0190 (7)	0.0344 (9)	0.0308 (8)	−0.0002 (6)	0.0039 (6)	−0.0024 (7)

### Geometric parameters (Å, °)

**Table:**

Ru1—N1	2.1165 (11)	N2—C4	1.3294 (18)
Ru1—S1	2.2853 (3)	N2—C3	1.3400 (19)
Ru1—Cl1	2.3381 (4)	N2—Na1^ii^	2.5224 (14)
Ru1—Cl4	2.3532 (3)	C1—C2	1.384 (2)
Ru1—Cl2	2.3555 (3)	C1—H1B	0.9500
Ru1—Cl3	2.3753 (3)	C2—C3	1.386 (2)
Cl2—Na1^i^	2.8888 (7)	C2—H2B	0.9500
Cl2—Na1	2.9576 (7)	C3—H3A	0.9500
Cl3—Na1	2.6988 (7)	C4—H4A	0.9500
S1—O1	1.4811 (11)	C5—H5A	0.9800
S1—C5	1.7708 (16)	C5—H5B	0.9800
S1—C6	1.7798 (16)	C5—H5C	0.9800
S2—O2	1.4969 (12)	C6—H6A	0.9800
S2—C7	1.7753 (18)	C6—H6B	0.9800
S2—C8	1.7806 (18)	C6—H6C	0.9800
Na1—O1^i^	2.2623 (12)	C7—H7A	0.9800
Na1—O2	2.3165 (12)	C7—H7B	0.9800
Na1—N2^ii^	2.5224 (14)	C7—H7C	0.9800
Na1—Cl2^i^	2.8888 (7)	C8—H8A	0.9800
O1—Na1^i^	2.2623 (12)	C8—H8B	0.9800
N1—C1	1.3451 (17)	C8—H8C	0.9800
N1—C4	1.3459 (18)		
			
N1—Ru1—S1	177.55 (3)	S2—O2—Na1	124.66 (7)
N1—Ru1—Cl1	88.93 (3)	C1—N1—C4	117.21 (12)
S1—Ru1—Cl1	88.676 (13)	C1—N1—Ru1	123.55 (10)
N1—Ru1—Cl4	90.96 (3)	C4—N1—Ru1	119.24 (9)
S1—Ru1—Cl4	89.645 (12)	C4—N2—C3	116.47 (13)
Cl1—Ru1—Cl4	92.407 (13)	C4—N2—Na1^ii^	115.02 (9)
N1—Ru1—Cl2	87.68 (3)	C3—N2—Na1^ii^	128.45 (10)
S1—Ru1—Cl2	91.887 (12)	N1—C1—C2	120.92 (13)
Cl1—Ru1—Cl2	91.791 (13)	N1—C1—H1B	119.5
Cl4—Ru1—Cl2	175.564 (12)	C2—C1—H1B	119.5
N1—Ru1—Cl3	88.51 (3)	C1—C2—C3	117.41 (13)
S1—Ru1—Cl3	93.885 (13)	C1—C2—H2B	121.3
Cl1—Ru1—Cl3	177.438 (13)	C3—C2—H2B	121.3
Cl4—Ru1—Cl3	87.525 (12)	N2—C3—C2	122.24 (13)
Cl2—Ru1—Cl3	88.217 (12)	N2—C3—H3A	118.9
Ru1—Cl2—Na1^i^	111.055 (16)	C2—C3—H3A	118.9
Ru1—Cl2—Na1	96.306 (15)	N2—C4—N1	125.70 (13)
Na1^i^—Cl2—Na1	107.313 (17)	N2—C4—H4A	117.1
Ru1—Cl3—Na1	103.076 (17)	N1—C4—H4A	117.1
O1—S1—C5	107.39 (8)	S1—C5—H5A	109.5
O1—S1—C6	105.24 (7)	S1—C5—H5B	109.5
C5—S1—C6	100.18 (9)	H5A—C5—H5B	109.5
O1—S1—Ru1	117.58 (4)	S1—C5—H5C	109.5
C5—S1—Ru1	112.02 (6)	H5A—C5—H5C	109.5
C6—S1—Ru1	112.80 (6)	H5B—C5—H5C	109.5
O2—S2—C7	106.88 (8)	S1—C6—H6A	109.5
O2—S2—C8	105.14 (7)	S1—C6—H6B	109.5
C7—S2—C8	98.53 (8)	H6A—C6—H6B	109.5
O1^i^—Na1—O2	97.03 (5)	S1—C6—H6C	109.5
O1^i^—Na1—N2^ii^	83.22 (4)	H6A—C6—H6C	109.5
O2—Na1—N2^ii^	88.83 (5)	H6B—C6—H6C	109.5
O1^i^—Na1—Cl3	168.75 (4)	S2—C7—H7A	109.5
O2—Na1—Cl3	94.20 (3)	S2—C7—H7B	109.5
N2^ii^—Na1—Cl3	97.50 (4)	H7A—C7—H7B	109.5
O1^i^—Na1—Cl2^i^	77.56 (3)	S2—C7—H7C	109.5
O2—Na1—Cl2^i^	106.49 (4)	H7A—C7—H7C	109.5
N2^ii^—Na1—Cl2^i^	156.63 (4)	H7B—C7—H7C	109.5
Cl3—Na1—Cl2^i^	98.81 (2)	S2—C8—H8A	109.5
O1^i^—Na1—Cl2	97.73 (4)	S2—C8—H8B	109.5
O2—Na1—Cl2	164.62 (4)	H8A—C8—H8B	109.5
N2^ii^—Na1—Cl2	97.16 (4)	S2—C8—H8C	109.5
Cl3—Na1—Cl2	71.032 (17)	H8A—C8—H8C	109.5
Cl2^i^—Na1—Cl2	72.685 (17)	H8B—C8—H8C	109.5
S1—O1—Na1^i^	138.58 (7)		
			
N1—Ru1—Cl2—Na1^i^	169.07 (4)	Na1^i^—Cl2—Na1—O2	89.29 (15)
S1—Ru1—Cl2—Na1^i^	−8.520 (18)	Ru1—Cl2—Na1—N2^ii^	87.18 (3)
Cl1—Ru1—Cl2—Na1^i^	80.213 (18)	Na1^i^—Cl2—Na1—N2^ii^	−158.42 (4)
Cl4—Ru1—Cl2—Na1^i^	−118.67 (16)	Ru1—Cl2—Na1—Cl3	−8.327 (15)
Cl3—Ru1—Cl2—Na1^i^	−102.351 (18)	Na1^i^—Cl2—Na1—Cl3	106.070 (19)
N1—Ru1—Cl2—Na1	−79.63 (3)	Ru1—Cl2—Na1—Cl2^i^	−114.397 (16)
S1—Ru1—Cl2—Na1	102.787 (16)	Na1^i^—Cl2—Na1—Cl2^i^	0.0
Cl1—Ru1—Cl2—Na1	−168.480 (16)	C5—S1—O1—Na1^i^	102.49 (12)
Cl4—Ru1—Cl2—Na1	−7.36 (16)	C6—S1—O1—Na1^i^	−151.41 (11)
Cl3—Ru1—Cl2—Na1	8.957 (16)	Ru1—S1—O1—Na1^i^	−24.88 (13)
N1—Ru1—Cl3—Na1	77.70 (4)	C7—S2—O2—Na1	−69.98 (10)
S1—Ru1—Cl3—Na1	−101.797 (18)	C8—S2—O2—Na1	−174.02 (8)
Cl1—Ru1—Cl3—Na1	80.2 (3)	O1^i^—Na1—O2—S2	6.47 (9)
Cl4—Ru1—Cl3—Na1	168.727 (18)	N2^ii^—Na1—O2—S2	89.50 (8)
Cl2—Ru1—Cl3—Na1	−10.026 (18)	Cl3—Na1—O2—S2	−173.06 (8)
N1—Ru1—S1—O1	−63.0 (8)	Cl2^i^—Na1—O2—S2	−72.58 (8)
Cl1—Ru1—S1—O1	−74.84 (6)	Cl2—Na1—O2—S2	−157.17 (10)
Cl4—Ru1—S1—O1	−167.26 (6)	S1—Ru1—N1—C1	−140.4 (7)
Cl2—Ru1—S1—O1	16.91 (6)	Cl1—Ru1—N1—C1	−128.53 (11)
Cl3—Ru1—S1—O1	105.25 (6)	Cl4—Ru1—N1—C1	−36.14 (11)
N1—Ru1—S1—C5	171.9 (8)	Cl2—Ru1—N1—C1	139.63 (11)
Cl1—Ru1—S1—C5	160.06 (7)	Cl3—Ru1—N1—C1	51.36 (11)
Cl4—Ru1—S1—C5	67.64 (7)	S1—Ru1—N1—C4	39.4 (8)
Cl2—Ru1—S1—C5	−108.20 (7)	Cl1—Ru1—N1—C4	51.30 (10)
Cl3—Ru1—S1—C5	−19.86 (7)	Cl4—Ru1—N1—C4	143.68 (10)
N1—Ru1—S1—C6	59.8 (8)	Cl2—Ru1—N1—C4	−40.54 (10)
Cl1—Ru1—S1—C6	47.92 (7)	Cl3—Ru1—N1—C4	−128.82 (10)
Cl4—Ru1—S1—C6	−44.49 (7)	C4—N1—C1—C2	−0.8 (2)
Cl2—Ru1—S1—C6	139.67 (7)	Ru1—N1—C1—C2	179.06 (11)
Cl3—Ru1—S1—C6	−131.99 (7)	N1—C1—C2—C3	1.8 (2)
Ru1—Cl3—Na1—O1^i^	6.4 (2)	C4—N2—C3—C2	−0.6 (2)
Ru1—Cl3—Na1—O2	−175.98 (3)	Na1^ii^—N2—C3—C2	176.46 (11)
Ru1—Cl3—Na1—N2^ii^	−86.60 (3)	C1—C2—C3—N2	−1.1 (2)
Ru1—Cl3—Na1—Cl2^i^	76.60 (2)	C3—N2—C4—N1	1.9 (2)
Ru1—Cl3—Na1—Cl2	8.427 (15)	Na1^ii^—N2—C4—N1	−175.64 (11)
Ru1—Cl2—Na1—O1^i^	171.27 (3)	C1—N1—C4—N2	−1.2 (2)
Na1^i^—Cl2—Na1—O1^i^	−74.33 (3)	Ru1—N1—C4—N2	178.99 (12)
Ru1—Cl2—Na1—O2	−25.10 (15)		

Symmetry codes: (i) −*x*+1, −*y*, −*z*+1; (ii) −*x*+1, *y*, −*z*+1/2.

## References

[bb1] Alessio, E., Balducci, G., Calligaris, M., Costa, G., Attia, W. M. & Mestroni, G. (1991). *Inorg. Chem.* **30**, 609–618.

[bb2] Alessio, E., Balducci, G., Lutman, A., Mestroni, G., Calligaris, M. & Attia, W. M. (1993). *Inorg. Chim. Acta*, **203**, 205–217.

[bb3] Alessio, E., Bolle, M., Milan, B., Mestroni, G., Faleschini, P., Geremia, S. & Calligaris, M. (1995). *Inorg. Chem.* **34**, 4716–4721.

[bb4] Anderson, C. & Beauchamp, A. (1995). *Inorg. Chim. Acta*, **233**, 33–41.

[bb5] Anderson, C. M., Herman, A. & Rochon, F. D. (2007). *Polyhedron*, **26**, 3661–3668.

[bb6] Antonarakis, E. S. & Emadi, A. (2010). *Cancer Chemother. Pharmacol.* **66**, 1–9. 10.1007/s00280-010-1293-1 PMC4020437 20213076

[bb7] Bruker (2007). *APEX2, *SADABS** and *SAINT* Bruker AXS Inc., Madison, Wisconsin, USA.

[bb8] Herman, A., Tanski, J. M., Tibbetts, M. & Anderson, C. M. (2008). *Inorg. Chem.* **47**, 274–280. 10.1021/ic062419h 18062685

[bb9] Iengo, E., Mestroni, G., Geremia, S., Calligaris, M. & Alessio, E. (1999). *J. Chem. Soc. Dalton Trans.* pp. 3361–3371.

[bb10] Jaswal, J. S., Rettig, S. J. & James, B. R. (1990). *Can. J. Chem* **68**, 1808–1817.

[bb11] Kostova, I. (2006). *Curr. Med. Chem.* **13**, 1085–1107. 10.2174/092986706776360941 16611086

[bb12] Sheldrick, G. M. (2008). *Acta Cryst.* A**64**, 112–122. 10.1107/S0108767307043930 18156677

[bb13] Silva, D. (2010). *Anti-Cancer Agents Med. Chem.* **10**, 312–323. 10.2174/187152010791162333 20380636

